# Risk factors for all-cause mortality during the COVID-19 pandemic compared with the pre-pandemic period in an adult population of Arkhangelsk, Russia

**DOI:** 10.1038/s41598-025-85360-0

**Published:** 2025-01-07

**Authors:** Ekaterina Krieger, Alexander V. Kudryavtsev, Ekaterina Sharashova, Olga Samodova, Vitaly A. Postoev

**Affiliations:** 1 Department of Community Medicine, UiT The Arctic University of Norway, Tromsø, 9037 Norway; 2 International Research Competence Centre, Northern State Medical University, Troitsky Ave., 51, Arkhangelsk, Russia 163069; 3 Department of Infectious Diseases, Northern State Medical University, Troitsky Ave., 51, Arkhangelsk, Russia 163069; 4 Department of Research Methodology, Northern State Medical University, Troitsky Ave., 51, Arkhangelsk, Russia 163069

**Keywords:** COVID-19, SARS-CoV-2, All-cause mortality, Risk factors, Russia, Infectious diseases, Biomarkers

## Abstract

We investigated and compared mortality rates and risk factors for pre-pandemic and pandemic all-cause mortality in a population-based cohort of men and women in Arkhangelsk, Russia. A prospective cohort study enrolled 2,324 participants aged 35 to 69 years between 2015 and 2017. All participants were followed up for all-cause deaths using the mortality registry. Mortality rates per 1000 person-years were calculated for men and women in the pre-pandemic and pandemic periods. Cox regression models were used to investigate demographic, lifestyle, and health characteristics associated with increased risk of death in both periods. During the pandemic, age-standardized all-cause mortality increased in women, but minor change was observed in men. Older age, smoking, and diabetes were associated with a higher risk of all-cause death in both periods and for both sexes. In women, higher risk during the pandemic was associated with obesity, angina, elevated cystatin C levels, and a history of COVID-19. In men, asthma and elevated hs-Troponin T levels increased the risk of death during the pandemic, while elevated hs-CRP and NT-proBNP levels were associated with higher risk in both periods. Targeted preventive interventions for men and women with specific risk factors can be implemented during potential future infectious disease outbreaks.

**Supplementary Information:**

The online version contains supplementary material available at 10.1038/s41598-025-85360-0.

## Introduction

On December 31, 2019, China reported several cases of severe pneumonia with an unknown origin. The causative agent was identified on January 7, 2020, and officially named severe acute respiratory syndrome coronavirus 2 (SARS-CoV-2)^1^. On March 11, 2020, the World Health Organization (WHO) declared the novel coronavirus (COVID-19) outbreak as a pandemic. The pandemic resulted in an overall increase in mortality, encompassing causes both directly related to COVID-19 and unrelated to the infection, which represent indirect consequences of the pandemic^2,3^.

Pre-existing chronic diseases heighten susceptibility to SARS-CoV-2 and the risk of COVID-19 complications, such as acute respiratory distress syndrome, sepsis, septic shock, and multiorgan failure^4^. Conversely, COVID-19 can worsen pre-existing chronic conditions, particularly cardiovascular diseases, by potentially damaging myocardial cells and being pathogenically linked to thrombovascular events^5–7^. Death resulting from any of these complications could be attributed to the virus, considered as a direct effect of the pandemic^8^.

The indirect consequences of the pandemic, including limited access to timely medical care due to overwhelmed healthcare systems, hindered routine and screening services, disproportionately affecting individuals with chronic diseases^2,9^. Additionally, these individuals could hesitate to seek medical care for non-urgent conditions out of fear of contracting SARS-CoV-2^10,11^. People older than 65 years, as well as those with chronic health conditions, were informed about the risks to their health associated with COVID-19 and officially advised to self-isolate^12^. The limited access to healthcare, coupled with reduced screening activities, mandatory isolation, and avoidance of seeking healthcare, could result in delayed diagnoses and treatment of conditions such as acute cardiovascular events, neoplasms, or the progression of chronic diseases, potentially contributing to the increased mortality^9,13,14^.

The mortality statistics, based on a single underlying cause of death, may not fully capture the complexity of factors or processes that lead to death in individuals with chronic diseases, including the role of the virus in this sequence^15,16^. Limited access to testing for laboratory confirmation of infection could also introduce misclassification of deaths related and unrelated to COVID-19, especially during the early stages of the pandemic^17,18^.

Given the multiple effects of different factors on mortality during the pandemic, counting deaths from all causes combined provides a more comprehensive approach to measuring the overall mortality effects of the pandemic, avoiding issues of attributing deaths specifically to COVID-19^2^. Assessment of the impact of demographic and lifestyle factors, pre-existing chronic diseases and the blood-based biomarkers on the risk of death during the pandemic compared with the pre-pandemic period could shed light on pathways of how the pandemic affected mortality, enabling targeted preventive efforts.

The study aimed to investigate and compare mortality rates and risk factors for pre-pandemic and pandemic all-cause deaths within a population-based cohort of adults in Arkhangelsk, Russia.

## Methods

### Study design and study population

A prospective cohort study was carried out involving participants of Know Your Heart (KYH) study of cardiovascular diseases, which has been described previously^19^. KYH study sample is a population-based cohort of 2357 participants (68% response rate) aged 35 to 69 years at enrolment from 2015 to 2017^19^. All participants underwent interviews conducted by trained interviewers, physical examinations and provided blood samples for further biomarker analysis. Follow-up period for a study participant started after the inclusion in the KYH study (later referenced as the baseline) and ended at the end of the COVID-19 pandemic (i.e. 5 May 2023) or at the date the participant died^20^. The total follow-up period was divided into the pre-pandemic period (date of KYH inclusion – 16 March 2020) and the pandemic period (17 March 2020–5 May 2023). Data collected at the baseline study were linked to the Arkhangelsk Regional Mortality Database (later referenced as the mortality registry). In this study, we included deaths from all causes among the participants recorded in the mortality registry during both periods.

Fifteen individuals with missing baseline data on any of the covariates were excluded, resulting in a pre-pandemic study sample of 2342 participants aged 35–69 years. All the study participants alive at the start of the pandemic in Arkhangelsk (17 March 2020) comprised the pandemic study sample of 2284 individuals aged 40–74 years. Data of the pandemic study sample were linked to the Federal Registry of COVID-19 Patients (later referenced as the COVID-19 case registry).

### Outcomes and covariates

For each death, data from medical death certificates were collected, including the date of death, immediate cause of death, related pathological conditions, underlying cause of death, external cause of death, and other contributing conditions in accordance with the International Classification of Diseases, 10th revision (ICD-10).

The baseline data were collected in 2015–2017 years by questionnaire, physical examination, and laboratory tests by following the KYH study protocol as described by S. Cook et al.^19^. As the KYH study was designed to investigate cardiovascular diseases, the baseline data included predominantly cardiovascular biomarkers.

The following data variables were considered as covariates in analyses for this study: demographic factors: age (years), sex (male/female), higher education (yes/no); lifestyle characteristics: current smoking (yes/no), hazardous alcohol drinking (score of ≥ 8 on the Alcohol Use Disorders Identification Test – AUDIT^21^) (yes/no)); self-reported doctor-diagnosed diseases: hypertension (yes/no), diabetes (yes/no), angina (yes/no), history of myocardial infarction (yes/no), heart failure (yes/no), asthma (yes/no), chronic bronchitis (yes/no), kidney disease (yes/no), liver disease (yes/no), neoplasms (yes/no); obesity (measured at physical examination as body mass index ≥ 30 kg/m^2^; yes/no); upper limits of the range of normal values for blood-based cardiac and metabolic biomarkers: lipid profile: total cholesterol ≥ 5.2 mmol/L (yes/no), low-density lipoprotein cholesterol (LDL-C) > 3.0 mmol/L (yes/no), high-density lipoprotein cholesterol (HDL-C) < 1.0 mmol/L for men and < 1.3 mmol/L for women (yes/no), triglycerides > 1.7 mmol/L (yes/no); a biomarker of hyperglycemia: glycated hemoglobin (HbA1C) ≥ 6.5% (yes/no); a biomarker of alcohol consumption and cholestasis: Gamma-glutamyl transferase (GGT) ≥ 40 U/L (yes/no); a biomarker of systemic inflammation: high-sensitivity C-reactive protein (Hs-CRP) ≥ 2 mg/L (yes/no); a biomarker of kidney dysfunction: Cystatin C ≥ 1.2 mg/L (yes/no); a biomarker of cardiac wall stretch (heart failure): N-terminal pro-b-type natriuretic peptide (NT-proBNP) ≥ 125 pg/mL (yes/no); a biomarker of heart damage: high-sensitivity Troponin T (Hs-Troponin T) ≥ 6 ng/L (yes/no). For the pandemic period, we also considered data on COVID-19 diagnosis (yes/no) from the COVID-19 case registry.

### Ethical approval

The study was conducted in accordance with the Declaration of Helsinki. The KYH study was approved by the ethics committees of the London School of Hygiene & Tropical Medicine, London, UK (approval number 8808, February 24, 2015) and Northern State Medical University (NSMU), Arkhangelsk, Russia (approval number 01/01–15, January 27, 2015). Ethical approval for the follow-up of the KYH participants was received from the ethics committee of NSMU (approval number 01/04–19, April 24, 2019). Ethical approval for the study of COVID-19 issues in the KYH cohort was obtained from the ethics committees of NSMU (approval number 01/02–21, February 17, 2021) and by the Regional Committee for Medical and Health Research Ethics, Norway (approval number 339397 received December 7, 2021).

All participants provided a written consent to disclose their medical and other health-related records for research purposes under the confidentiality condition. The data linkage between KYH data, the COVID-19 case registry, and the regional mortality registry was performed by the Arkhangelsk Regional Medical Information Analytical Center (MIAC) in accordance with the NSMU-MIAC confidentiality agreement based on the informed consents obtained from the participants, legal and ethical approvals. The participants were anonymized by the randomly assigned unique ID numbers and the following data linkage were based on these depersonalized IDs, which has led to no personal identifiers in the analyzed dataset.

### Statistical analysis

Participants characteristics were presented as medians with first and third quartiles for continuous variables, absolute numbers and percentages for categorical variables. Accordingly, Mann–Whitney U-test and Pearson’s chi-squared test were used for comparing groups on continuous and categorical characteristics.

All-cause and cause-specific (defined by the ICD-10 chapters) mortality rates per 1000 person-years were calculated for men and women in both the pre-pandemic and pandemic periods. Mortality rates for the pandemic period were directly age-standardized to the age distribution of the study population in the pre-pandemic period using 5-year bands. Mortality rates were presented with 95% confidence intervals (CIs).

Age-adjusted mortality ratios in the pandemic period were estimated as hazard ratios (HR) derived from Cox proportional hazards regression models of the studied death outcomes, where period (1 = pandemic period, 0 = pre-pandemic period) and age in years were entered as covariates. Person-months of observation in the pre-pandemic (date of KYH inclusion − 16 March 2020) and pandemic (17 March 2020-5 May 2023) periods were used as time variables in Cox models for the pre-pandemic and pandemic periods, respectively. When modelling each of death outcomes, interaction of the period with sex, likewise other interactions considered in this study, was assessed by comparing models with and without the interaction term using the likelihood ratio test. Based on the identified interaction of the period with sex, further analyses were stratified by sex. We also used Cox models to investigate the factors associated with a change (increase or decrease) in the risk of pre-pandemic and pandemic deaths. For each independent variable, interaction with the study period was assessed. The effect estimates were reported as HRs adjusted for demographic (age, higher education) and lifestyle (smoking, hazardous drinking) variables with the corresponding 95% CIs. All the statistical analyses were performed in Stata version 17.0 (StataCorp, College Station, TX, USA). A p-value < 0.05 was considered statistically significant.

## Results

The studied population comprised 980 men (41.8%) and 1362 women (58.2%). During a median follow-up period of 6.5 years (6.0; 7.0), a total of 150 deaths occurred among the study participants. In the pre-pandemic period, 45 men and 13 women died, leaving a sample of 935 men and 1349 women for the pandemic period. A total of 56 men and 36 women died in the pandemic period.

### Characteristics of the study participants

The median age was 53 (44; 61) years at the time of inclusion in KYH study (at baseline) and 57 (48; 65) years at the onset of the pandemic (Supplementary Table 1). Men were more likely to be smokers and hazardous drinkers compared to women. Higher proportions of women compared to men had obesity, diabetes, heart failure, asthma, chronic bronchitis, kidney disease, liver disease, and neoplasms. Conversely, more men than women reported having prior myocardial infarction.

Women exhibited a higher proportion of elevated total cholesterol and decreased HDL-C levels, while increased triglycerides were more prevalent among men (Supplementary Table 2). Men were also more likely to have elevated levels of GGT and Hs-Troponin T, whereas elevated levels of NT-proBNP were more commonly observed among women.

During the pandemic, one third of men and women had COVID-19 documented in the case registry. Women had a higher proportion of infections (35.6%) than men (31.2%), *p* = 0.031.

### Mortality rates

**Figure 1:**
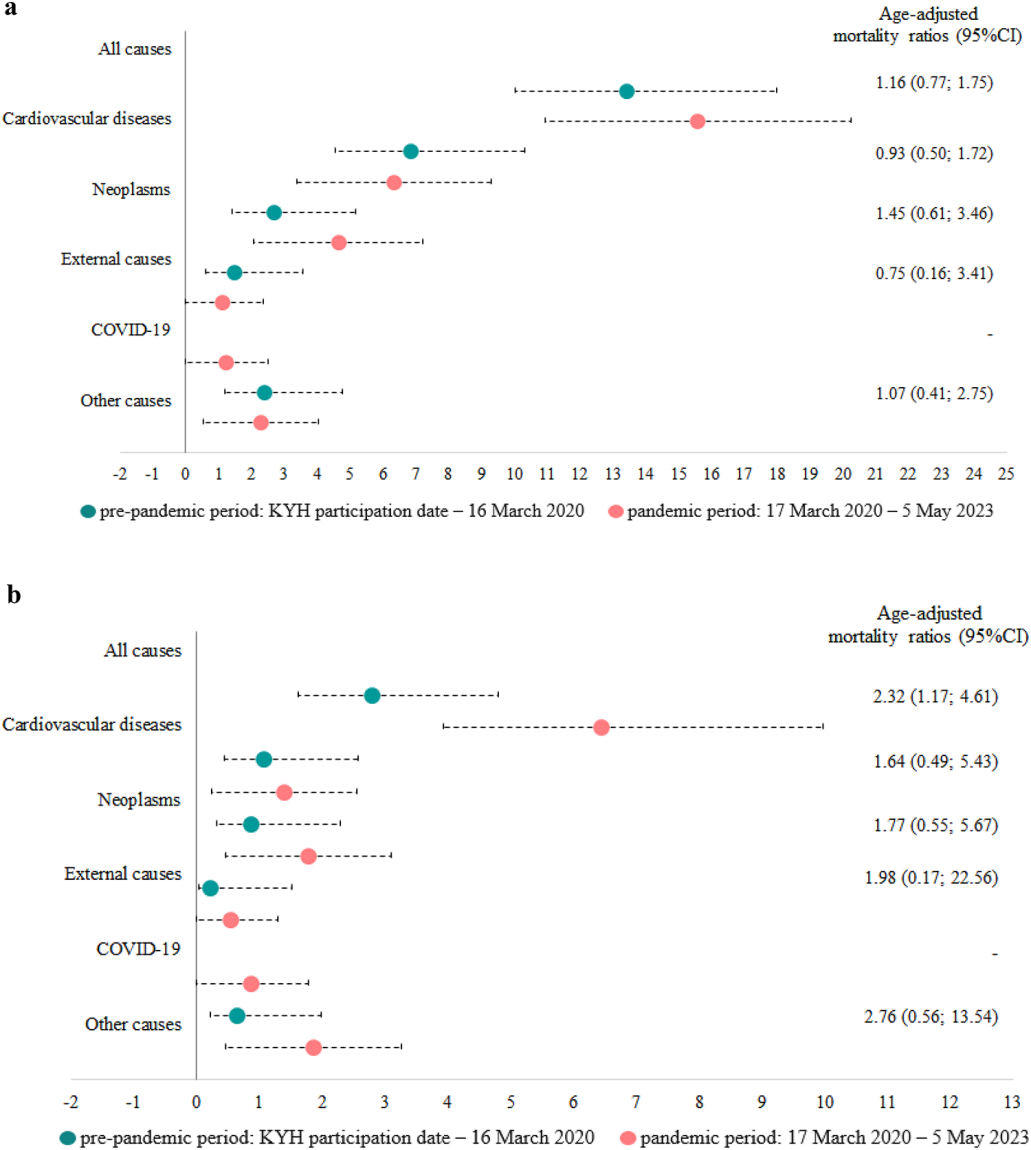
Mortality rates per 1 000 person-years with 95% confidence intervals (95% CI) during the pre-pandemic and pandemic periods for men (a) and women (b). Estimates for the pandemic period were age-standardized to the age distribution of the study population during the pre-pandemic period. Age-adjusted mortality ratios (95% CI) represent hazard ratios derived from Cox regression models for the studied mortality outcomes, with the period (1 = pandemic period, 0 = pre-pandemic period) and age in years entered as covariates.

The mortality rates were higher in men compared to women in both periods (Fig. 1).

In women, age-standardized all-cause mortality rates increased from 2.79 per 1000 person-years in the pre-pandemic period to 6.45 per 1000 person-years in the pandemic period (a 2.32-fold increase), but they did not change significantly in men (*p* = 0.047 for the interaction between sex and the study period). In the pandemic period, neoplasms were the leading cause of death in women, followed by cardiovascular diseases, whereas the opposite pattern was observed in the pre-pandemic period. In men, cardiovascular diseases were the leading cause of death in both periods, followed by neoplasms. Mortality rates from COVID-19 as underlying cause of death were similar in men and women.

### Risk factors for pre-pandemic and pandemic all-cause deaths

**Figure 2:**
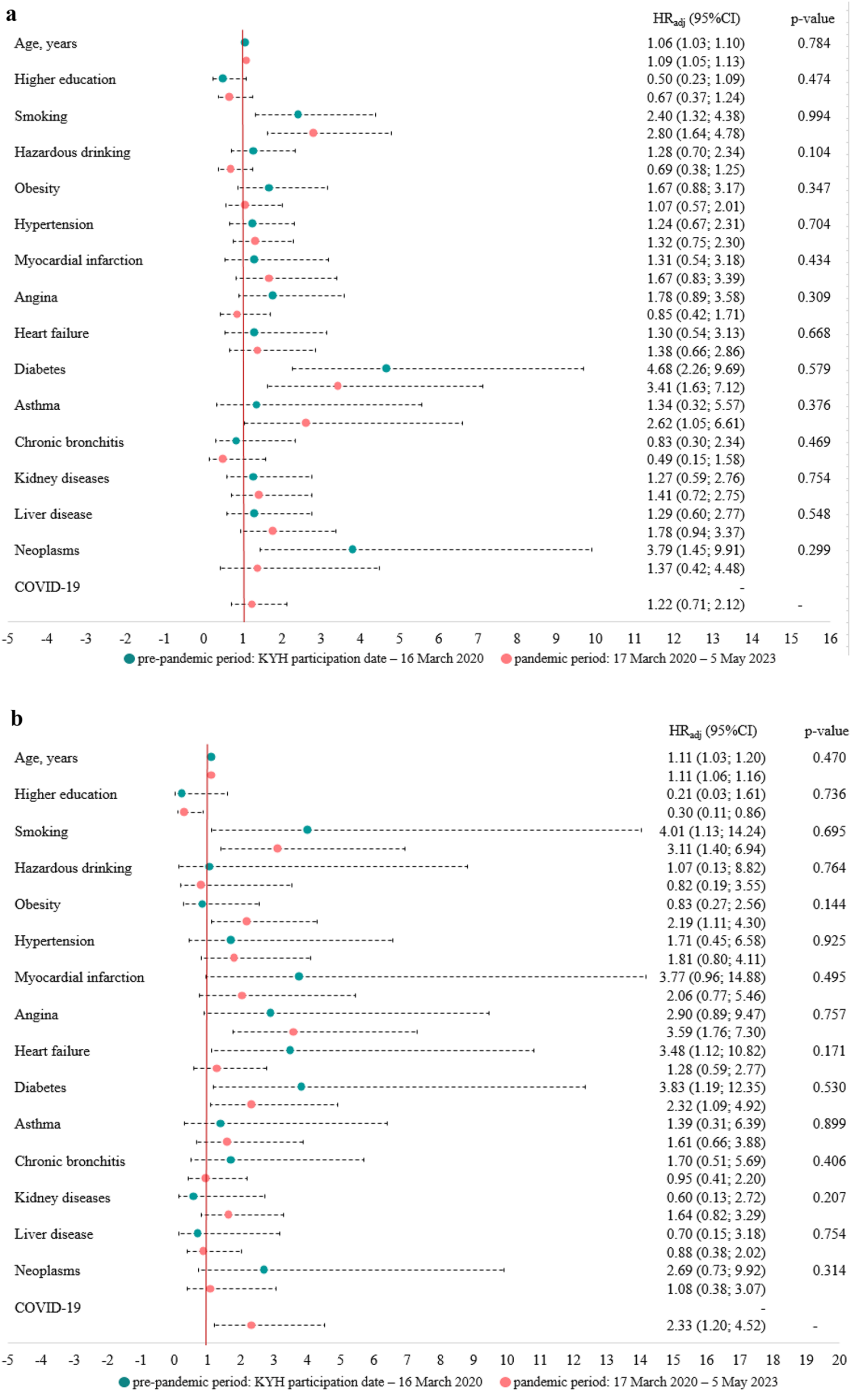
Cox regression models describing the associations between demographic and health-related factors and all-cause mortality during the pre-pandemic and pandemic periods in men (a) and women (b). HR_adj_ (95% CI) - hazard ratios with 95% confidence intervals, adjusted for demographic factors (age, higher education) and lifestyle factors (smoking, hazardous drinking). The p-value is for the interaction with the time period. All factors, except for COVID-19 (new coronavirus disease), were collected at baseline (2015–2017). Age is presented as at baseline for the pre-pandemic period and as at 17 March 2020 for the pandemic period.

After adjustment for demographic and lifestyle factors, older participants, smokers, and those with a self-reported diagnosis of diabetes had a significantly higher risk of all-cause death in both sexes in both periods (Fig. 2). In men, neoplasms were associated with increased the risk of all-cause death only in the pre-pandemic period, whereas asthma increased the risk only in the pandemic period. In women, self-reported heart failure was associated with increased risk of death only in the pre-pandemic period, while obesity, and angina – only in the pandemic period. Higher education was associated with a decreased risk of death during the pandemic in women. A history of COVID-19 was associated with a higher risk of all-cause mortality in women, but not in men. There were no significant interactions between the study period and demographic, lifestyle, and health characteristics in their effects on all-cause mortality.

**Figure 3:**
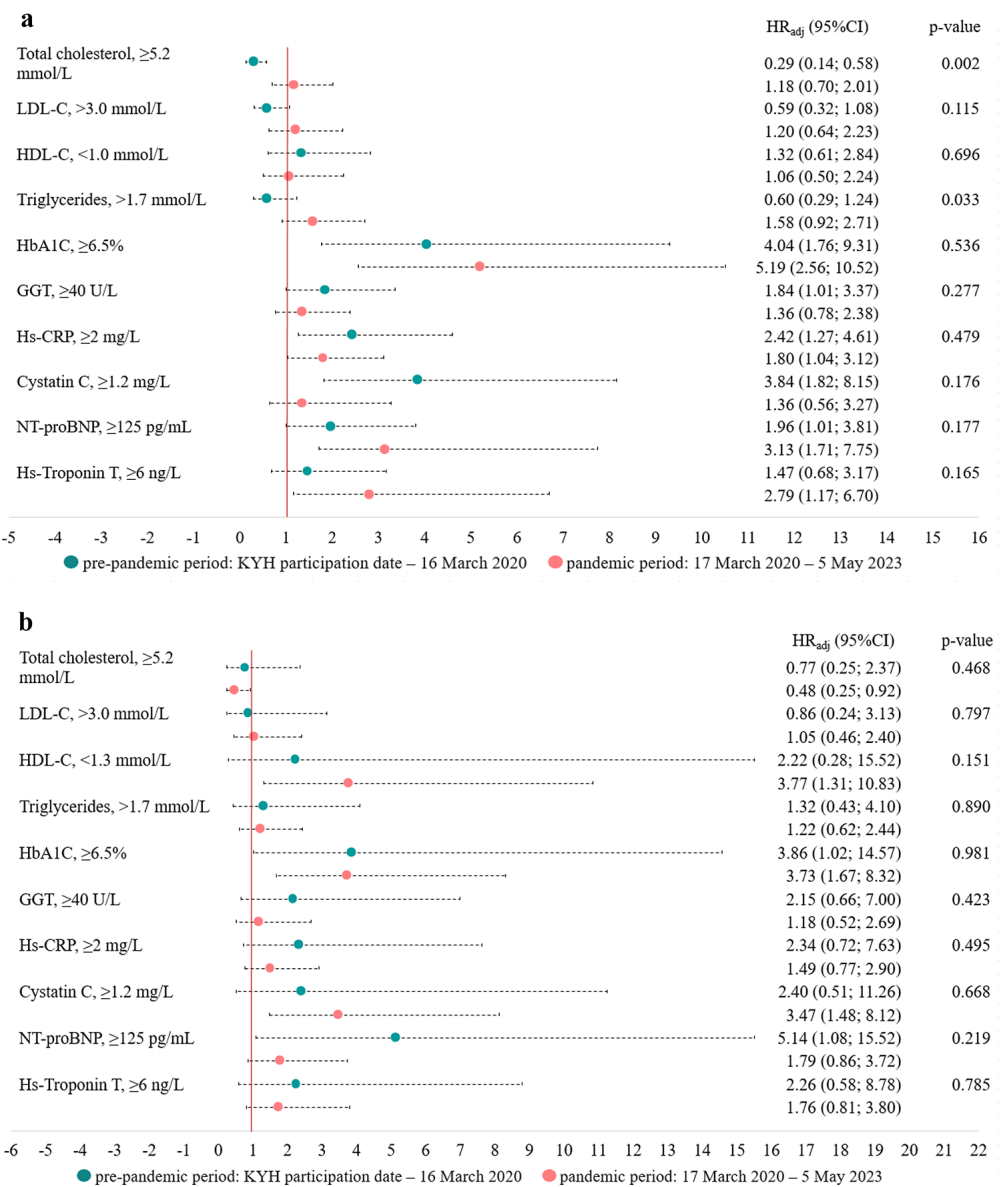
Cox regressions describing the associations between blood-base biomarkers and all-cause mortality during pre-pandemic and pandemic periods in men (a) and women (b). HR_adj_ (95% CI) - hazard ratios with 95% confidence intervals, adjusted for demographic factors (age, higher education) and lifestyle factors (smoking, hazardous drinking). The p-value is for the interaction with the time period. All biomarkers were measured at baseline (2015–2017). LDL – low-density lipoprotein cholesterol, HbA1C – glycated hemoglobin, GGT – Gamma-glutamyl transferase, Hs-CRP – high-sensitivity C-reactive protein, NT-proBNP – N-terminal pro-brain natriuretic peptide, Hs-Troponin T – high-sensitivity Troponin T.

 Elevated levels of HbA1C increased risk of death in both sexes during both periods (Fig. 3). In men, during the pre-pandemic period, elevated levels of Hs-CRP, Cystatin C, and NT-proBNP were associated with an increased risk, while elevated levels of total cholesterol were associated with a lower risk of all-cause deaths. During the pandemic period, men had increased risks of death associated with elevated levels of Hs-CRP, NT-proBNP and Hs-Troponin T. The strength of the association between elevated NT-proBNP and risk of death increased during the pandemic period, although there was no significant interaction with the study period, *p* = 0.177. The protective effect of elevated cholesterol decreased during the pandemic period (p for interaction with period = 0.002), while the effect of triglycerides increased (p for interaction with period = 0.033), although the effects of triglycerides were not significant in any of the periods. In women, elevated NT-proBNP levels were associated with a higher risk of pre-pandemic deaths, while elevated Cystatin C levels increased the risk during the pandemic (Fig. 3).

## Discussion

The age-standardized mortality rates were higher in men during both periods, although the increase in mortality rates during the pandemic period was more pronounced in women than in men. Cardiovascular diseases and neoplasms were the leading causes of death in both periods and for both sexes. During the pandemic period, mortality from neoplasms exceeded mortality from cardiovascular diseases in women, while cardiovascular diseases remained the leading cause of death in men. Mortality from COVID-19 as the underlying cause were similar in both sexes. Compared with the pre-pandemic period, during the COVID-19 pandemic, women had an increased risk of death associated with obesity, angina, elevated cystatin C levels, and a history of COVID-19, while men had an increased risk of death associated with asthma, elevated biomarkers of cardiovascular risk.

Previous studies have shown that during the COVID-19 pandemic, all-cause excess mortality, the difference between observed and expected deaths, was highest in Russia among European countries^3,22,23^. In Russia, women experienced higher excess mortality than men, while in most countries excess mortality was higher in men^24^. A population-based study involving 63 countries showed substantial sex differences in age-standardized COVID-19 mortality rates, which were greater than those for all-cause mortality, with higher mortality rates in men than in women^25^. On the contrary, women were more susceptible to COVID-19 than men in countries such as India, Nepal, Slovenia, and Vietnam^26,27^.

Several studies have reported an increase in mortality from cardiovascular diseases and neoplasms during the COVID-19 pandemic, suggesting that the increase in deaths is largely due to indirect effects of the pandemic, such as delayed diagnosis and treatment^28–30^. However, other authors have suggested that the increase in deaths from cardiovascular diseases and neoplasms may be primarily due to undetected deaths related to COVID-19^31^.

 To our knowledge, this is the only study in Russia comparing mortality rates and risk factors for pre-pandemic and pandemic all-cause mortality in a population-based cohort. In our study, mortality among women increased significantly during the pandemic, with a history of COVID-19 associated with a higher risk of all-cause death. Notably, factors associated with increased mortality in women – such as obesity, angina, and elevated cystatin C levels – are also risk factors for death from COVID-19, supporting that women may be more vulnerable than men to complications directly related to the virus^32–34^. Except for asthma, the increased risk of death in men during the pandemic was associated with elevated biomarkers of cardiovascular risk. The lack of differences in COVID-19 mortality rates between the sexes may be due to an underestimation of the role of the virus and misclassification of COVID-19 deaths as non-COVID-19 deaths. Deaths from virus-related thrombovascular events may have been recorded as deaths from cardiovascular causes, or deaths in COVID-19 patients with advanced cancer may have been classified as deaths from neoplasms, especially when testing availability was limited^35^.

Given that the age of participants at the start of the pandemic ranged from 40 to 74 years, deaths in the study sample were relatively seldom and most could be considered premature. Therefore, some well-known risk factors were not associated with the risk of death during the two relatively short study periods.

 Higher education had a protective effect on the risk of death during the pandemic in women, but not in men. The association between higher education and mortality during the pandemic is complex and may involve factors such as employment status, work arrangements, social interactions, adherence to non-pharmaceutical interventions, healthcare-seeking and testing behaviors, and vaccination intentions^36,37^.

Our study found a positive association between smoking and mortality risk in both study periods for both sexes, consistent with evidence linking smoking to mortality from both COVID-19 and non-COVID-19 causes^2,38–40^. Despite recent findings suggesting an association between hazardous alcohol drinking and increased risk of death during COVID-19 pandemic due to poor health status and comorbidities, our study did not observe such an association^41–43^.

Women with angina and men with elevated levels of Hs-CRP, Hs-Troponin T and NT-proBNP were at higher risk of death during the pandemic period, consistent with other research^6,27,44,45^. Alongside the increased risk of encountering severe COVID-19 and life-threatening thrombovascular complications of COVID-19 during the acute phase of illness and for several months following infection^5,34,46^, individuals with cardiovascular diseases experienced the indirect effects of disrupted healthcare services and delayed diagnostics and treatment^28,29^.

Patients with pre-existing cardiovascular disease had a higher risk of death during the pandemic than those with pre-existing chronic respiratory disease, even though SARS-CoV-2 primarily affects the respiratory system^47^. In our study, asthma appeared to be associated with death in men during the pandemic, contradicting findings published by other authors^48^. Sex differences in the association may be related to poorer asthma control in men compared with women, although there is no clear consensus on the association between sex and treatment compliance^49^. Another study found that inhaled corticosteroids used to treat asthma may reduce the severity of COVID-19, with lower rates of hospitalization and death reported among users^50,51^.

 Obesity, a well-established risk factor for severe COVID-19, increased the risk of death in women during the pandemic, consistent with previous research^2,52,53^. Women in our study may be more vulnerable to complications directly related to the virus, possibly due to the higher prevalence of obesity compared to men^54^. Obesity exacerbates SARS-CoV-2-induced inflammation, leading to cytokine storms and increased risk of severe illness and mortality^32,55,56^.

Elevated total cholesterol levels were paradoxically associated with a lower risk of all-cause death in the pre-pandemic period for men and in the pandemic period for women, possibly due to the use of lipid-lowering medications. Other authors have reported that in younger individuals, elevated total cholesterol levels increase the risk of death, whereas in older individuals with multiple comorbidities, total cholesterol levels decrease due to the use of lipid-lowering medications while the risk of death increases, leading to a negative association between elevated total cholesterol levels and death^57^. The effects of biomarkers influenced by medication use must be interpreted with caution, as medication status is likely to have changed over time.

Neoplasms were associated with death in men shortly after enrollment and before the pandemic. Since baseline data on neoplasms were not updated, the pandemic sample included long-term cancer survivors diagnosed over 5 years ago, potentially underestimating the impact of neoplasms on pandemic mortality.

 We found no significant associations between chronic kidney disease or chronic liver disease and mortality in both study periods, despite previous research suggesting potential associations between these conditions and COVID-19-related mortality^27,58,59^.

Elevated Cystatin C levels were associated with an increased risk of death in women in the pandemic. Although a biomarker of kidney dysfunction, elevated levels of cystatin C are also associated with an increased risk of cardiovascular events related to endothelial dysfunction due to atherosclerosis^60,61^ and are correlated with severe COVID-19 and COVID-19-related death^33,62,63^. Although elevated cystatin C levels may be associated with obesity, recent research has shown that obesity does not affect the prognostic value of cystatin C for estimating the risk of adverse outcomes^64^.

The study contributes to understanding how COVID-19 affected premature mortality in men and women, as well as identifying the risk factors associated with death during the COVID-19 pandemic compared to the pre-pandemic period in the Russian adult population. We analyzed all-cause mortality rather than cause-specific mortality data to indirectly assess the impact of the COVID-19 pandemic on overall mortality, accounting for potential misclassification between COVID-19 and non-COVID-19 deaths. In our analysis, we consider the timing of deaths to assess whether pre-existing chronic diseases and blood-based biomarkers exhibit varying associations with subsequent mortality over time. The relatively high response rate of 68% in the KYH study suggests that the study sample is representative of the population of Arkhangelsk, aged 35–69 years at the baseline.

 Our study has several limitations. The baseline characteristics of the study participants, particularly blood-based biomarkers, were collected at a single time point more than five years before the pandemic period, and they might have changed over time. The cross-sectional Epidemiology of Cardiovascular Diseases and their Risk Factors in Regions of the Russian Federation (ESSE-RF3) study, conducted in 2021, provided mid-follow-up updates for half of the KYH participants and allowed comparisons of key demographic and health characteristics and selected biomarkers between the KYH and ESSE-RF3 datasets for the same participants. We found no differences in educational level, proportion of current smokers, or hazardous alcohol drinking. The prevalence of hypertension, obesity, diabetes (including HbA1c), and neoplasms was higher in ESSE-RF3, reflecting age-related increases and disease progression. The proportion of individuals with elevated LDL-C decreased, likely due to the initiation of lipid-lowering treatment. Therefore, single measurements of health characteristics and biomarkers may not reflect long-term exposure, and the association of these characteristics with mortality risk should be interpreted with caution due to the possibility of misclassification.

The completeness of the registry data may have influenced the study results. Although the COVID-19 case registry provided accurate information on cases detected by the health care system, limited testing capacity early in the pandemic may have resulted in missed cases. The lack of data on dropouts is another limitation. Some individuals may have relocated, making it no longer possible to register their outcomes. The proportion of unaccounted dropouts likely increased over time, resulting in a higher number of dropouts during the pandemic period. Consequently, they may have been misclassified as alive, potentially resulting in underestimated mortality rates during the pandemic period. Cause-specific mortality rates may also be affected by the accuracy of information in death certificates. The relatively small number of deaths analyzed was due to the relatively young age of the cohort, which may limit the interpretation of the results regarding factors associated with risk of death.

## Conclusions

During the COVID-19 pandemic, mortality rates remained higher in men than in women, with cardiovascular disease being the leading cause of death. There was a two-fold increase in premature mortality in women, but a minor change in men. The increase in mortality in women during the pandemic could be explained by the effects of obesity, angina and elevated Cystatin C levels (indicating kidney dysfunction), with those without higher education being more vulnerable. Along with elevated biomarkers of cardiovascular risk, asthma emerged as a factor associated with increased risk of death during the pandemic in men. Targeted preventive measures for women and men with specific risk factors can be implemented during potential future infectious disease outbreaks.

## Electronic supplementary material

Below is the link to the electronic supplementary material.

Supplementary Material 1

## Data Availability

Data from the Know Your Heart Study are available upon reasonable request after contacting Alexander V. Kudryavtsev at alex.v.kudryavtsev@yandex.ru. See data access regulations and instructions at https://metadata.knowyourheart.science (Accessed on 7 June May 2024). All data requests will be guided by protecting of personal information, confidentiality agreement with participants, and their informed consents.
