# Supplementary material for: Risk factors for all-cause mortality during the COVID-19 pandemic compared with the pre-pandemic period in an adult population of Arkhangelsk, Russia
Source: Sci Rep. 2025 Jan 7;15:1050. doi: 10.1038/s41598-025-85360-0 (PMC11706959; doi:10.1038/s41598-025-85360-0)
Supplement: Supplementary file 1 — Supplementary Material 1 [file 41598_2025_85360_MOESM1_ESM.docx]

**Supplementary material**

| Characteristics^a^ | Men, N=980 | Women, N=1362 | p^b^ |
| --- | --- | --- | --- |
| Age, years | 53 (44-61) | 53 (44-61) | 0.684 |
| Higher education | 334 (34.1) | 510 (37.4) | 0.094 |
| Smoking | 338 (34.5) | 207 (25.2) | <0.001 |
| Hazardous drinking | 373 (38.1) | 91 (6.7) | <0.001 |
| Obesity | 238 (24.3) | 460 (33.8) | <0.001 |
| Hypertension | 454 (46.3) | 676 (49.6) | 0.114 |
| Myocardial infarction | 69 (7.0) | 47 (3.5) | <0.001 |
| Angina | 133 (13.6) | 192 (14.1) | 0.717 |
| Heart failure | 87 (8.9) | 181 (13.3) | 0.001 |
| Diabetes | 64 (6.5) | 132 (9.7) | 0.006 |
| Asthma | 39 (4.0) | 109 (8.0) | <0.001 |
| Chronic bronchitis | 86 (8.8) | 189 (13.9) | <0.001 |
| Kidney diseases | 124 (12.7) | 305 (22.4) | <0.001 |
| Liver disease | 142 (14.5) | 263 (19.3) | 0.002 |
| Neoplasms | 27 (2.8) | 105 (7.7) | <0.001 |

Supplementary table 1. Baseline demographic, lifestyle and self-reported health characteristics of the study population stratified by sex, N=2342. ^a^Me – median, Q1 – the first quartile; Q3 – the third quartile for age, N (%) – for all other characteristics. ^b^Pearson’s chi-squared test for categorical parameters, Mann–Whitney U-test for continuous characteristics.

| Characteristics ^a^ | Men, N=980 | Women, N=1362 | P^b^ |
| --- | --- | --- | --- |
| Total cholesterol, ≥5.2 mmol/L, N (%) | 498 (50.8) | 810 (59.5) | <0.001 |
| LDL-C, >3.0 mmol/L, N (%) | 737 (75.2) | 1035 (76.0) | 0.661 |
| HDL-C, <1.0 mmol/L for men and <1.3 mmol/L for women, N (%) | 134 (13.7) | 342 (25.1) | <0.001 |
| Triglycerides, >1.7 mmol/L, N (%) | 305 (31.1) | 334 (24.5) | <0.001 |
| HbA1С, ≥6.5% | 45 (4.6) | 68 (5.0) | 0.655 |
| GGT, ≥40 U/L, N (%) | 349 (35.6) | 216 (15.9) | <0.001 |
| Hs-CRP, ≥2 mg/L, N (%) | 405 (41.3) | 571 (41.9) | 0.772 |
| Cystatin C, ≥1.2 mg/L, N (%) | 47 (4.8) | 50 (3.7) | 0.178 |
| NT-proBNP, ≥125 pg/mL, N (%) | 255 (26.0) | 513 (37.7) | <0.001 |
| Hs-Troponin T, ≥6 ng/L, N (%) | 672 (68.6) | 632 (46.4) | <0.001 |

Supplementary table 2. Baseline blood-based biomarkers characteristics in the study population stratified by sex, N=2342. ^a^N (%) – for all the characteristics. ^b^Pearson’s chi-squared test.
